# Exploring GP work in areas of high socioeconomic deprivation: a secondary analysis

**DOI:** 10.3399/BJGPO.2021.0117

**Published:** 2021-11-10

**Authors:** Marianne McCallum, Sara MacDonald

**Affiliations:** 1 General Practice and Primary Care, Institute of Health and Wellbeing, University of Glasgow, Glasgow, UK

**Keywords:** inequalities, continuing professional development, qualitative research, multimorbidity, workforce

## Abstract

**Background:**

There is a GP workforce crisis, particularly in areas of high socioeconomic deprivation where levels of multimorbidity and social complexity are higher than in areas of low socioeconomic deprivation. How this impacts GP work, and how GPs manage workload has not been fully explored.

**Aim:**

To explore GP work in areas of high socioeconomic deprivation and the strategies GPs employ, using Corbin and Strauss’s framework on managing chronic illness as an analytical lens.

**Design & setting:**

Secondary analysis of qualitative in-depth interviews with GPs working with populations experiencing high levels of socioeconomic deprivation.

**Method:**

Secondary analysis of in-depth interviews with GPs working in areas of high socioeconomic deprivation (*n* = 10).

**Results:**

All three types of work defined by Corbin and Strauss (everyday, illness, and biographical) were described, and one additional type: emotional (work managing GPs’ own emotions). The context of socioeconomic deprivation, increased multimorbidity plus social complexity (’multimorbidity plus’), influenced GP work. Healthcare systems and self-management strategies did not meet patients’ needs, which meant the resulting gap created extra everyday work, often unrecognised (which was a source of frustration). GPs also described taking on ’illness work’ for patients who were either overwhelmed or unable to do it. Some GPs described biographical work, asserting their professional role against demands from patients and other professionals. Work aligning with personal values was important in sustaining motivation; for example, being part of a strong team and having outside professional interests appeared to build resilience.

**Conclusion:**

GPs working in areas of high socioeconomic deprivation experience different types of work from those working in areas of low socioeconomic deprivation; much of which is unrecognised and not resourced. Current strategies to reduce burnout could be more effective if the complexity of different types of work was addressed. In addition, personal values, practice teams, and outside professional interests all need to be supported.

## How this fits in

Understanding GP work and how best to support it is critical, particularly in areas of socioeconomic deprivation where multimorbidity and social complexity are higher than in areas of low socioeconomic deprivation. GPs carry out four specific types of work (everyday, illness, biographical, and emotional). Socioeconomic deprivation influences this work, which is often not recognised by the wider health system, and is under-resourced. Current workforce strategies focus only on illness work. The present article suggests that supporting all types of work may be beneficial, particularly for practices working in the context of high socioeconomic deprivation.

## Introduction

Multimorbidity (the presence of ≥2 long-term conditions) is a major challenge to global health systems,^1^ and is associated with poorer outcomes and increased healthcare utilisation.^2^ The challenge posed by multimorbidity is not simply increasing prevalence,^3^ but the paucity of evidence on how best to manage it.^1,4^ Multimorbidity disproportionally affects communities experiencing high socioeconomic deprivation, where it starts at an earlier age.^3^

Additionally, in areas of high socioeconomic deprivation, GP consultation rates are higher, covering more problems in less time, with no additional resource.^5–7^ In this context, psychosocial problems are more common, referrals more complex,^8^ and patients often struggle to manage their illnesses.^9^ Consultations demonstrate lower levels of patient enablement and higher levels of GP stress.^6,7^

This mismatch of resource and need exemplifies Tudor Hart’s inverse care law, which is as relevant today as it was 50 years ago.^10,11^ In the UK, universal primary care limits the impact of market forces and removes most financial barriers to care, but more equitable distribution of resources, including in primary care, could more effectively mitigate health inequalities.^11,12^ However, this must be set in the context of challenges in recruitment and retention, particularly in deprived areas.^13^

There is a GP workforce crisis in the UK,^14^ with an urgent need to reduce GP burnout and stress.^15^ This article focuses specifically on practices in Scotland: while there are some important differences in how GP care is delivered between the devolved nations, the primary care system is broadly comparable and experiencing similar strains across the UK. Current strategies to manage this crisis focus on reducing GPs’ clinical workload, and shifting aspects of illness management to other health professionals.^16^ Understanding if, and how, socioeconomic deprivation impacts GP work, and what strategies GPs use to manage that work, is critical to ensure primary care services are effective, well equipped, resourced, and supported.

Previous explorations of the nature of work in the context of chronic illness have typically focused on the work of patients. Corbin and Strauss‘s *Managing* *c* *hronic* *i* *llness* *at home: three lines of work* (summarised in Table 1) proposed that managing chronic illness required three types or ‘lines’ of work: illness work (work required to manage illness); everyday work (organisation and coordination of various types of work necessary to operationalise any plans of action); and biographical work (the work required in defining and making an identity). These three lines of work require to be balanced, defined as ‘relative equilibrium‘. Instability of relative equilibrium requires the enactment of strategies to maintain balance and adjust to changes in context resulting in ‘unending work‘.^17^ The authors are not aware of these propositions being applied in the context of professional work, but given that the management of chronic illness is at the core of general practice work, Corbin and Strauss’s propositions offer a potentially useful analytical framework for exploring different types of GP work, and whether socioeconomic deprivation influences the work that is carried out.

**Table 1.** Summary of Corbin and Strauss’s *Managing* *c* *hronic* *i* *llness at* *h* *ome:* *t* *hree* *l* *ines of* *w* *ork* ^17^

**Lines of work:** the different types of work required to manage chronic illness	**Illness work:** the work required to manage their illness. **Everyday work:** organisatino and coordination of various types of work necessary to operationalise any plans of action. **Biographical work:** the work required in defining and making an identity.
**Managing work:**strategies patients used to manage their workloads	**Conditional motivation:** biographical schemes (work aligning with personal values) that helped motivate the patient to cope with their illness. These were hopeless if not attainable.**Management in process:** strategies patients used to maintain relative equilibrium. **Calculating resources:** as all three types of work were in competition for limited resources patients needed to calculate and allocate resources, as well as moving resources flexibly as required. **Maintaining fluid boundaries** in the context of division of labour, recognising shifts in roles because of illness, and ensuring flexibility in how tasks are divided. **Ongoing articulation of work:** planning and coordinating work, which will continue to vary so constant reassessment is required. **Mutual sustaining:** importance of others to sustain commitment to the work, the importance of alignment, which is more than communication but is the process by which people ‘mutually align their actions towards the performance of some work‘.

The aim of this study was to explore GP work in areas of high socioeconomic deprivation using Corbin and Strauss’s framework on managing chronic illness as an analytical lens.

## Method

### Study design

The original study^18^ employed qualitative interviews to explore GP training in areas of high socioeconomic deprivation. During inductive analysis several themes about GP work in this context appeared. A qualitative secondary supra analysis^19^ of the in-depth interviews (*n* = 10) was performed, drawing on Corbin and Strauss‘s^6,7^ work.

### Setting

A sampling framework (described in detail in the original article^18^) identified potential practices serving populations experiencing high socioeconomic deprivation. Within this group potential practices of different sizes were intentionally sampled from a variety of geographical locations across Scotland. Fifteen practices were originally contacted, 11 responded and 10 GPs were able to go onto full interview. Table 2 summarises the main characteristics of the practices and sex of the interviewees.

**Table 2.** Summary characteristics of study practices and demographics of interviewees (with comparison, where relevant, to Scottish average)

Characterstic	*n* ^a^
Participants	10 (5 trainers, 5 non-trainers)
**Location (** **r** **egion of deanery)**	
West	5
East	2
South-East	2
North	1
**Practice size**	
Average practice size	5620
Scotland average	5710
Range in practices interviewed	2827–9118
**Deprivation**	
Proportion of practice in the most deprived quintile (2015), %	63.61
(Scotland average), %	16.2
Range in practices interviewed, %	25.6–87.9
Average deprivation score^b^	4.54
Scotland average	3.11
Range in practices interviewed	3.95–4.81
**Age of interviewee, years**	
>50	4
41–50	5
30–40	1
**Sex** **of interviewee**	
Female	8
Male	2

a Unless otherwise stated. ^b^Deprivation score weighted by the proportion of the practice population in each of the five deprivation quintiles: 1 (most affluent)–5 (most deprived). For example, practice eight had a lower proportion of patients in quintile 1 but had a higher score as almost all their patients lived in postcodes in the two most deprived quintiles.

### Data collection

In-depth interviews were carried out using a semi-structured topic guide (see Supplementary Appendix S1) by one researcher. The interviews were all conducted between January and May 2017. They lasted between 45 and 60 minutes, and were audiorecorded, transcribed verbatim, and anonymised.

### Data analysis

The inductive thematic analysis from the original study has been published elsewhere.^18^ A secondary supra analysis analysed the transcripts using Corbin and Strauss’s work on managing chronic illness as a theoretical lens.^17,20^ A coding framework, characterised by Corbin and Strauss’s themes, was applied to the transcripts:

- Everyday work
- Illness work
- Biographical work
- Conditional motivation
- Relative equilibrium
  - Calculating resources
  - Maintaining fluid boundaries of labour
  - Ongoing articulation of work
  - Mutual sustaining.

A line-by-line analysis, using the theoretical framework, on two of the transcripts determined the applicability of the framework. It fitted well with the data. The authors discussed the fit of the themes in the remaining transcripts and the framework was then applied to all transcripts using NVivo software (version 11) to manage data.

### Ethics

All interviewees provided full informed consent (see Supplementary Appendix S2) for their anonymised transcripts to be held for 5 years and used for further research.

## Results

**Figure 1.**
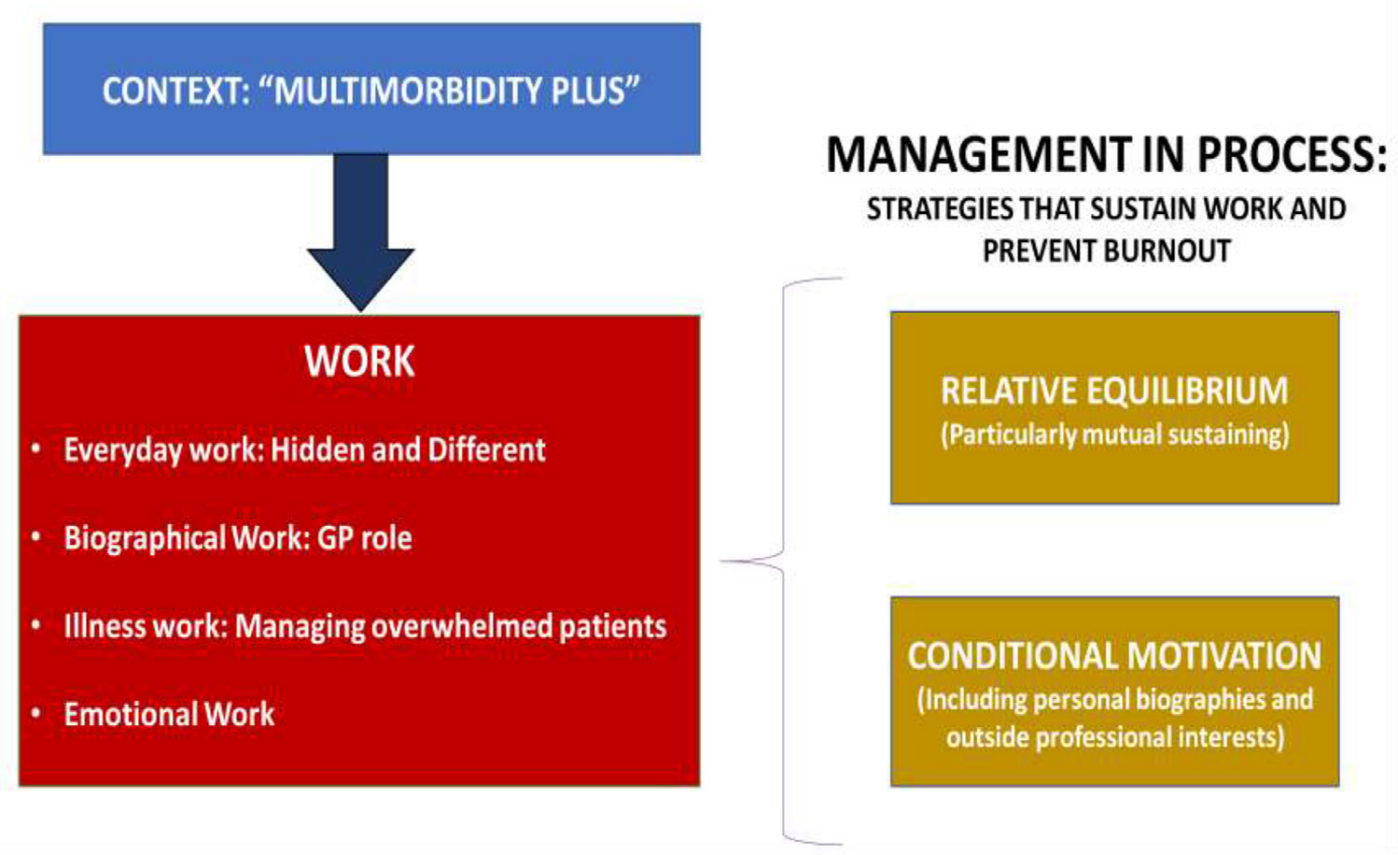
Summary of themes, subthemes, and their relation to one another

Figure 1 summarises the final key themes (context, work, and management in process) developed from the original framework, and how they interrelate to one another.

### Context: multimorbidity plus

All GPs interviewed identified key characteristics of the communities they worked in that impacted their work: higher prevalence of early multimorbidity; patients managing the impact of poverty, alcohol, and drug addiction; vulnerable families; migrant populations; and patients for whom life was chaotic.

They described how the experience of multiple disadvantage limited efforts to improve health outcomes for patients. All recognised the increased burden of multimorbidity at an earlier age, which they felt distinguished their work from those serving more affluent populations. While increased medical complexity made this harder to manage, balancing the management of increased medical complexity in the context of social complexity was particularly difficult. One characterised this as ’multimorbidity plus’:

*’...* *it is known there is more multimorbidity, I think often the multimorbidity is what’s talked about rather than the* *multimorbidity plu* *s* *perhaps a greater chance of having difficulties with literacy plus greater chance of having an ethnic element coming in, it is the several layers* *.’* (GP6, female [F], aged 41–50 years)

### Work

#### Everyday work: hidden and different

Managing this ‘multimorbidity plus‘ (Box 1) was perceived to result in work unlike that undertaken by colleagues who worked in other less deprived areas:

Box 1 **Summarising how the context of socioeconomic deprivation created different everyday work**

Multiple migrant populations

- *‘* *...* *we’re trying to count non-English speaking groups and we think it is in excess of 5% in our surgery and our surgery looks slightly United Nations from the outside.’* (GP9, male [M], aged >50 years)
- *’* *I didn’t mention also the challenges within a deprived practice are increasingly* *...* *immigrant populations. I appraise as a GP and I appraise some of the outlying, more of the suburban outlying practices, and just coming down to the fact that several GPs in those kind of areas have said they’ve never used the telephone interpreting service. I use it, earlier this week, I counted up I had used in over 50% of my consultations. Which instantly builds in the time factor because everything is slowed down when you are interpreting and translating* *...* *and and a lot of these people are very new to this country there are massive health issues, language barriers, culture barriers, all sorts of things which again is this raft of hidden work that is never recognised so yes we need far far far more GP time in a practice like this and* *...* *than perhaps elsewhere.’* (GP5, F, aged >50 years)

Low health literacy

- *’* *You’re dealing with people who can’t read and write, and I just saw a man this morning who’d been to the* *u* *rology clinic with quite significant problems, and they sent him away and said you have to fill in a bladder chart. I’d already discussed doing a bladder chart with him and he said he wouldn’t do it because he can’t read, but they’d given him this thing and they hadn’t questioned it and he’d just decided not to go back because, you know* *,* *”* *I can’t do that because I canny read.”’* (GP7, F, aged 41–50 years)
- *’* *Yes I mean a huge number of our patients can’t read or write, so the spoken word, you still have to use very simple language with them, so it’s a different way of communicating. You can’t just hand a leaflet out. They’ve not been reading on Google, they’ve not been looking at different things, they will come in getting us to be their advocates, and to fill in forms for them because they can’t do that. And that’s very different from being a GP in an affluent area, even recently I was helping a friend out in her practice in* [Town X]*, it was a completely different concept where people wanted a bit more self-management or have the money to go and pay for a physio for their back pain, or aren’t labourers so their back pain isn’t impacting as much on their work, so it was nice seeing a different aspect of what I do here.’* (GP8, F, aged 30–40 years)

Managing chaotic lives

- *’...* *the decision making that goes on with running a practice in an area where there is social complexity and where there is more expression of emotions than you might otherwise get when people are …* *under the influence of alcohol and under the influence of drugs, when people are struggling to get through life, struggling to financially manage then there is* *…* *is naturally more expression of emotion and I think I can say that is just relating to the demographic rather than just the personality* *.’* (GP6, F, aged 41–50 years)
- *’...* *when you see them you then have this range of issues* *…* *this range of psychological, social* *,* *and physical needs that you do your best to address in the contact you have because you know that they may go away and the chances of catching them again are small* *.’* (GP2, F, aged >50 years)

*’* *I think all GPs are always very very busy, but we have to ask ourselves are we busy with the same things and what does the NHS want to focus on? So* *...* *we have on the one hand probably the worried well that keep affluent practices very busy, and probably also the fear of making mistakes there. And they have the high elderlys* *but we also know from the sample point of the Scottish allocation formula the deep-end practices are underfunded in relatively to the* *...* *morbidities, and also I think we have to spend much more time in safety netting, patient education or sometimes opting for, because our patients are struggling to understand the safety netting.’* (GP1, F, aged >50 years)

This different everyday work was described as ’hidden’ by GPs. Health system configurations, with an emphasis on self-management and self-referral, failed to consider the everyday realities of patients’ lives, further disadvantaging an already disadvantaged group. The additional GP work required to fill this gap was absorbed into routine everyday work, unrecognised and under-resourced, by the wider health system:

*’* *I think, as I said earlier, a lot of the increased workload in this kind of context is completely hidden and therefore unmeasurable and therefore unrecognised and I think it’s a dream to think there will ever be any additional funding to recognise that kind of work, it’s never going to happen* *.’* (GP5, F, aged >50 years)

All the GPs found work overwhelming at times, and the current workload was felt unsustainable. Several were quite fatalistic about whether this could ever change given the wider issues with GP shortages:

*’...* *we’re all busy and we’re all sort of treading water just to stop ourselves from drowning so it’s a workload issue. And we’ve got this very difficult chicken and egg scenario, that if there were more GPs we would all feel less stressed, but we’re now all too stressed to train more GPs* *… What could they do to help us? I don’t know. Invent more hours in the day and fewer patients. It’s workload, it’s too busy. We just don’t have the headspace and the time and capacity any more to do it.’* (GP10, F, aged 41–50 years)

#### Biographical work: GP role

Biographical work to assert professional identity was described across the interviews. Some GPs described a persistent struggle to define what they saw as their role as a GP against pressures from patients and other professionals:

*’* *...* *in our patient population I think one of the main challenges is that they don’t, there’s always this feeling that we’re not quite doing what we were trained to do. We were trained in the orthodox this is how illness presents, these are the symptoms, this is the diagnosis, you make your management plan and off you go. Whereas I think in a deprived population so many of the presentations are they just don’t follow those pathways, you know patients are polysymptomatic, much of their illness behaviour comes out of mental health problems, it’s a complexity about teaching in that kind of context* *.’* (GP5, F, aged >50 years)

Constantly asserting professional identity, including refusing requests (for example, letters for housing applications), was an ongoing source of daily biographical work. Other GPs, however, viewed managing social complexity, and attendant patient advocacy, as key parts of their role; they embraced these tasks and absorbed them into their professional identity and therefore spent less time grappling with biographical work. For these GPs embracing this wider role appeared to give them a sense of peace and purpose as it aligned with personal values about equity and justice. However, the downside of this stance was that absorbing such roles created additional routine work (for example, doing the referral for patients who struggle to use the self-referral system in context of low literacy, building links with the third sector):

*’* *And any self* *-* *referrals are usually very difficult, for example* *...* *physio self* *-* *referral because quite often our patients don’t know how to fill the forms out* *…* *So in most of the cases if it’s something that should be seen urgently it’s not picked up by the physios because our patients just can’t fill it out properly* *.’* (GP1, F, aged >50 years)

#### Illness work: managing overwhelmed patients

GP participants felt that the increased medical and social complexity impacted on their patients’ outcomes and ability to self-manage. For many patients, overwhelmed by the work of managing long-term illness, decision making became the responsibility of the primary care team. GPs in deprived areas felt that many of their patients preferred a more doctor-centred approach:

*’It’s interesting, I look at the college* [RCGP] *… this whole concept of shared decision making and, I actually think you consult in a different way in a deprived practice and I think your decision making* *…* *You do some of it with chronic disease but actually it’s a lot more, doctor centred* *...* *to use that old model because it’s your patients would look at you like you’ve got two heads and I’m stupid. When I do shared decision making, they’re like* *“what you asking me for?* *”’* (GP8, F, aged 30–40 years)

#### Emotional work

One area of work that did not feature in Corbin and Strauss’s framework was emotional work. Emotional work characterises the work of GPs to manage the impact of observing, and at times being unable to offer help in the difficult situations patients experience. The impact of this was significant for most of the GPs:

*’...* *there is, I think there is a greater potential in your day to be emotionally knocked when working in a deprived area* *… and that that has a drain on you, your energy and resources. So obviously there’s lots of emotional things happen in general practice but I think it is difficult sometimes to realise what awful lives some people lead and you have to share that with them.’* (GP6, F, aged 41–50 years)

### Management in process

#### Conditional motivation

Corbin and Strauss recognised the importance of conditional motivation via biographical schemes: activities that aligned with their values and sense of identity. Evidence of this was found in the analysis of conditional motivation. The impact of wider values, and the alignment of identity and purpose in GPs’ work environments (particularly in the context of reducing inequality and making a difference), provided ongoing motivation to continue their work:

*’* *…* *think as medics we should be aware of where the health* *care problems are greatest and I don’t think it’s good enough to just take yourself off to some leafy suburb where life’s a bit easier. Because that’s not where the problems are and it’s not looking to see, if you know we want to understand about health problems and disease within society, then we need to look at the whole picture and the impact of the way in which we run our society and the effect its having on our population and we all have a responsibility to that.’* (GP2, F, aged >50 years)

All recognised the impact of wider social determinants of health on patients’ lives and framed their work, and what could be achieved within that context. This is in keeping with Corbin and Strauss’s findings that successful biographical schemes must be achievable. While recognising that what could be achieved was limited by wider factors, the potential to make a difference in people’s lives, provided powerful motivation and resilience:

*’* *And those lovely moments where you actually, really can make a real, that satisfaction that you can make a real difference in somebody’s life.’* (GP8, F, aged 30–40 years)

Similarly, being involved in additional professional activities, which allowed them time to reflect and remind themselves of their early motivation for opting to work in general practice was important. All the GPs were involved in external activities (such as undergraduate or postgraduate training, appraising, local medical committees, or part of the Scottish GPs at the Deep End^21^ steering group). These roles fostered resilience and reduced burn out. They offered a break from clinical work and provided professional tasks that could be completed in contrast to the uncertainty and unending demand from clinical work:

*’* *And the task is contained when you’ve finished teaching, the kind of teaching I do, you turn off the computer and the lights and it’s finished, done, you drive away and that session is done. There is nothing following it, no one is going to phone, you know there’s no letters, no one is going to become ill overnight. So it’s great, it’s brilliant, it’s like liberation. Likewise, with appraising it’s a contained task, once you’ve done it you’ve done it and that’s it, and I find that really helpful that at least part of my work is like that.’* (GP 5, F, aged >50 years)

#### Relative equilibrium

Examples of GPs and wider practice teams articulating work, allocating resources, and having fluid boundaries were all seen within interviews. As in Corbin and Strauss’s work, this was in response to changes in resource, or to relieve recognised areas of pressure.

However, of the strategies identified by Corbin and Strauss, the need for mutual sustaining was uniformly recognised as vital. GPs spoke of how a supportive flexible team, with a shared ethos, was essential in managing workloads (Box 2).

Box 2 **The importance of team ethos**

- *’...* *you need colleagues that are going to be very much committed to stepping in and committed to having discussions because people’s lives are very complex* *.’* (GP6, F, aged 41–50 years)
- *’...* *but if you want to do it properly in my opinion you have to get a bit involved, and that has a cost, and if you can share that with colleagues then it doesn’t break you.’* (GP6, F, aged 41–50 years)
- *’...* *you can have, when you are dealing with patients who potentially are very chaotic at their core. And there can be that, almost like, at times, an agenda conflict. At times the interface can be a hostile one. Not always at all, but there can be a challenge there. If your team were broken and you were trying to deal with all of the issues, you just couldn’t do it. So you need your team to be with you because you just never know who’s the one who might be having a bad day.’* (GP8, F, aged 30–40 years)
- *’* *I don’t think any of us are feeling we absolutely have to do this because otherwise we’d go broke. I think there is a feeling with all of us that we’re kind of privileged to be able to do the kind of medicine that we do here, and if the income isn’t as good as it is down the road then you know, that’s fine* *.’* (GP5, F, aged 41–50 years)
- *’...* *you speak to each other at the end of the morning, at the end of the afternoon. You make sure that you are talking about things that are difficult to deal with, that’s with the fellow GPs, and then with your staff* *.’* (GP2, F, aged >50 years)

## Discussion

### Summary

The analysis suggests that, in the context of high socioeconomic deprivation, general practice comprises several lines of ’work’, stretching far beyond the biomedical management of illness. Neither the hidden everyday work, nor the additional emotional work, were perceived to be recognised or resourced within the wider healthcare system. While current strategies for managing workload primarily focus only on reducing illness work,^22^ the research suggests that strategies that support and resource the additional and varied nature of work are required. Supporting factors that appear to build resilience are necessary to support GPs, and prevent burnout, particularly in areas of high socioeconomic deprivation.

The analysis extends Corbin and Strauss’s framework on managing chronic illness and offers a useful analytical lens to understand professional management of chronic illness in areas of high socioeconomic deprivation. While the context of high socioeconomic deprivation brought unique workload issues, much of the work described by GPs was thought to align well with GPs in other communities. It is likely, therefore, that Corbin and Strauss’s framework would be useful to explore professional work across other contexts.

### Strength and limitations

One strength of this study is that participating GPs, despite coming from a range of practices (in terms of location and size), were consistent in their description of work and the challenges they encountered. It is believed the findings regarding GP work reflect socioeconomic deprivation, not just local geography. Additionally, using Corbin and Strauss’s well-established *Managing* *c* *hronic* *i* *llness* *at home* *: three lines of work* ^17^ as an analytical lens provided a framework to interpret the findings, which increases and extends its applicability in settings outside of patient experience, and provides a basis for further research exploring health professionals’ work in other contexts.

Limitations include the small sample and the recognition that GPs working in other areas have different challenges. In addition, these interviews are a secondary analysis and 4 years old; therefore, they don’t take into account the impact of the pandemic, which has had a significant impact on GP and wider NHS workload. The authors also did not have information on GP workload (for example, number of patients per GP and number of consultations per day), which would be expected to impact the participant’s experience of work. Future work may wish to look at exploring this, although, as the results indicate, how to meaningfully measure GP workload is difficult; for example, factors such as social complexity, which significantly impacts experience of work, are hard to quantify, and not routinely measured. Further research with GPs in other contexts is required to understand GP workload and wider applicability of this analytical lens. Nevertheless, many of the GPs had worked in other contexts, and concluded that work in areas of high socioeconomic deprivation presented additional challenges.

### Comparison with existing literature

The findings are in keeping with wider literature on general practice in deprived areas, in relation to the pressure of increased multimorbidity,^3,7,9^ increased prevalence of psychosocial problems,^3,7,9^ impact of social complexity,^3,7,9,23–25^ and issues created by multimorbidity at a younger age.^3,26^ Similar to the layers of ’multimorbidity plus’ described here, previous work has framed multimorbidity, in the context of socioeconomic deprivation, as the combination of physical, social, and psychological problems,^25^ and the difficulty managing with a lack of emotional or social resources.^25,27^

Patient capacity to self-manage is key, yet is often not taken into account.^28^ Self-management strategies may be unachievable when patients lack capacity and resource.^9,25,27^ The GPs in this study identified systemic barriers, and inflexible systems that failed to account for differences in capacity to carry out tasks.

Previous work has illustrated variation in how GPs in deprived areas view their role.^25,29^ For instance, GPs who view unhealthy behaviours as a product of wider social factors, in contrast to those who viewed them as choices, had a wider definition of their professional role, including patient advocacy.^25,29^ The work demonstrates that those GPs also required less biographical work. Debate regarding the GP role continues, particularly in the context of increasing workloads.^22,30,31^ This includes how the expertise of general practice to define and manage problems can be recognised and whether GPs should be ’consultants’ in general practice.^31^ The new Scottish GP contract envisions GPs as expert generalists who manage the most complex patients.^22^ The findings show that complexity must be extended to incorporate social, as well as other medical factors.

Emotional work^32^ or emotional labour^33^ is recognised in the wider literature, predominantly in a nursing context.^34^ In the context of GPs working in socioeconomic deprivation there is, to the authors’ knowledge, no specific research on emotional work, although managing patients’ distress has a recognised emotional impact.^25^ Emotional work can be supported by self-reflection in peer groups; social and wider organisational, or workplace support; and the maintenance of professional boundaries.^32–35^

Current policy promotes shared decision making,^36^ yet GPs report making decisions for overwhelmed patients and being ’doctor-centred’ in response to patient preference. Patient-centred care (PCC) has no official definition; the literature describes a wider more holistic philosophy,^37^ including communication, partnership, recognition of wider context, and focusing beyond illness to healthy lifestyles.^37^ Concepts of PCC vary by professional lens (particularly between managers and clinicians).^38^

Understanding what builds GP resilience, particularly in areas of high socioeconomic deprivation where professional burnout is higher,^15^ is crucial given the current workforce crisis. While much research focuses on individual characteristics, and supporting individuals,^39^ the importance of teamwork is critical.^39,40^ In the context of socioeconomic deprivation, where *’* *resilience is enacted through teams not individuals* *’*,^40^the value of flexibility for health professionals working in this context is recognised,^40^ as is motivation,^41^ for professional wellbeing and reducing burnout.^40,41^

### Implications for research and practice

Current strategies to manage GP workload and stress have focused on reducing illness work; for example, by shifting specific parts of routine work to other members of the team such as pharmacists or physiotherapists. The findings suggest that this approach may have limited success if other areas of ‘work’ are not also addressed. First, everyday work that was described as ’hidden’ by participating GPs needs to be understood, quantified, and adequately resourced.

Second, it was found that emotional work contributes significantly to GP workload. Supporting practice teams, allowing regular time for practice reflection and regular personal supervision may be of value to help GPs manage this work. In addition, resourcing GPs to be involved in other interests outside their clinical work (for example, teaching or cluster work) appears likely to be of benefit, particularly if it aligns with underlying values.

Managing multimorbidity creates work, but the social complexity that creates ’multimorbidity plus’ significantly increases workload. Efforts to reduce GPs’ work should include staff who can manage social complexity; for example, welfare rights workers.^42^ Recognition of the impact of personal views for motivation should also be acknowledged. Initiatives that allow GPs to explore personal values and motivation, and how to align these with their professional work, may help prevent burnout.^40,41^

This study was a secondary analysis of existing interviews that sought to explore whether Corbin and Strauss’s framework on managing chronic illness has utility beyond patienthood, and if it was a useful analytical lens to understand GP work. While the findings demonstrate the applicability of the analytic lens, since the original data were collected the GP workforce experience has changed, particularly owing to the COVID-19 pandemic. Repeating this work in the current context (the ongoing pandemic) would allow deeper understanding of the impact that COVID-19 has had on GP workload. In addition, wider sampling to understand GP work in different contexts (for example, affluent and rural populations) is needed, as it is expected there are different workload implications in different contexts, which may require different resourcing. This is essential information to ensure proper resourcing of primary care teams and to fully understand GP work demand in different contexts.

In conclusion, Corbin and Strauss’s framework on managing chronic illness provides a useful theoretical lens to understand GP work in areas of high socioeconomic deprivation and may be useful in other contexts. GPs in deprived areas describe several types of work, some of it hidden; strategies are needed that support all these types of work. In addition, initiatives that enhance team ethos, personal motivation, and support outside professional activities could further build resilience in the GP workforce.
